# Development and characterization of moringa oleifera fruit waste pod derived particulate cellulosic reinforced epoxy bio-composites for structural applications

**DOI:** 10.1016/j.heliyon.2022.e09755

**Published:** 2022-06-20

**Authors:** Isiaka Oluwole Oladele, Gabriel Seun Ogunwande, Anuoluwapo Samuel Taiwo, Senzeni Sipho Lephuthing

**Affiliations:** a Department of Metallurgical and Materials Engineering, Federal University of Technology, PMB, 704, Akure, Ondo State, Nigeria; b Department of Metallurgy, University of Johannesburg, South Africa; c Enhanced Composites and Structures Centre, School of Aerospace, Transport, and Manufacturing, Cranfield University, United Kingdom; d Centre for Nanomechanics and Tribocorrosion, School of Metallurgy, Chemical and Mining Engineering, University of Johannesburg, Johannesburg, South Africa

**Keywords:** Moringa fruit pod, Environmental concern, Eco-friendly, Epoxy bio-composites, Green material, Structural application

## Abstract

The desire for environment-friendly materials and sustainability has brought a paradigm shift in the way engineers and the entire material research community thinks while attempting to develop new material, particularly for engineering applications. This study is carried out to underscore the suitability of particulate moringa oleifera fruit pod (MOFP) reinforced epoxy bio-composites on selected properties for structural applications. The dried waste fruit pods were processed as calcined and pulverized fruit pod particulates, respectively. Their respective bio-composites were developed by blending the selected materials in predetermined proportions using the open mould processing method. The MOFP particles were characterized with SEM/EDS and XRD while mechanical and wear properties of the developed bio-composites were evaluated. The results showed that the pulverized MOFP reinforced epoxy bio-composites showed improved properties than the calcined MOFP bio-composites in most of the properties considered. This was noticed to be due to the presence of more elemental constituents and at higher proportions in pulverized particles than in the calcined particles. It was discovered that 15 wt.% pulverized MOFP reinforced epoxy bio-composites gave about 67.9%, 28.7%, 8.8%, and 8.8% enhancement and with a value of 70.2 HS, 39.02 MPa, 198.4 MPa, and 753.28 MPa in hardness, flexural strength, flexural modulus, and tensile modulus, respectively to emerge as the reinforcement content with the optima properties. Based on the findings, MOFP particles reinforced epoxy-based biocomposites can be used in applications where stiffness and high strength are not essential requirements; packaging applications; in electrical component applications such as circuit boards, and cables due to their low thermal conductivity.

## 1 Introduction

The European Union (EU) officials, sectors, and especially the automotive industry are all interested in green materials and green materials technologies. Moreover, the quest for eco-friendly strategies and procedures through green science, engineering, and technology has been at the center of global initiatives toward sustainable development and environmental protection by social movements [1]. Scientists and industrialists look to nature, particularly natural fibres, for feasible greener answers to these concerns. Their global abundance and ease of access to agro-waste are driving ongoing research into greener materials [2, 3]. Natural fibres' application and applicability in the development of greener solutions for the manufacturing, consumption, and disposal of automotive products have scientists and politicians optimistic [4]. The financial logic of investing in green materials research and manufacturing is also influenced by widely held consumer needs. Much academic and corporate research is focused on finding new ways to manufacture environmentally friendly chemicals and materials for a number of purposes. Natural fibres are frequently viewed as a panacea for a variety of environmental issues, including end-of-life (ELV) vehicles, trash minimization, and economic development projects [5].

Polymer-based materials have recently become the preferred materials for a variety of applications. Today, more advanced polymer materials are being produced on a regular basis as an alternative to other materials, especially in areas where polymers were previously thought to be unsuitable. Most of the constraints of polymers are addressed in the formation of composite materials, making these possible. Scientists and researchers are also working on adaptability to beneficial environmental influences [6].

One of the ways by which polymers are being modified to suit human needs and fit for engineering applications is found in the development of bio-based composites. Natural fiber-based bio-composites are increasingly being used in items such as car interiors, electronic devices, the building, and construction sector, packaging, and so on [7, 8]. However, natural fibers are highly heterogeneous, and their properties vary across the globe. This property variation depends on the geographical location, vegetation, age, species, parts/types of the plant or animal, feeds/nutrition, method of extraction, and mode of application in the matrix among others. Therefore, this diversification has made the development of bio-composites an area of continuous research interest. As a result, there is a high demand for research into the promising and new features of sustainable natural fibres as a bio-reinforcement in polymer bio-composites for various applications. Several natural fibres have been researched to improve the mechanical properties of reinforced and filled polymer bio-composites, including jute, banana, sisal, bagasse, bamboo, grass, flax, lufa, coir, cotton, wool, hair, feather, and many more [9]. However, recently in Nigeria, the more readily available natural fibers like pawpaw [10], banana, plantain, coconut, and sisal [2, 11] from plants are being researched. The results from the studies revealed a potential use of these cellulosic fibers as reinforcement materials in polymers.

Another readily and abundantly available vegetable reinforcement material is obtainable from Oleifera Moringa fruit pods. The plant is commonly referred to as the ben oil tree. It is commonly farmed in various parts of Nigeria and other tropical African countries. The plant thrives in a wide range of soil types, preferring well-drained, slightly alkaline, sandy, or loamy soils [12]. It is regarded as one of the world's most useful plants because practically all its parts can be utilized for food, traditional medicine, and industrial applications. The outer portion of the fruit (usually called a pod) is tough and rough, which is a waste product and almost in all cases discarded indiscriminately in the environment. It is high in fiber and is discarded once its economic worth has been realized. Certain benefits of natural fibers are availability, manufacturing ease, and less offensive than synthetic fibers in terms of production equipment, sustainability, and biodegradability [13].

Various studies have been conducted to determine the applicability of various components of the Moringa oleifera tree. The mechanical, physical, and tribological behaviours of polymer bio-composites based on natural fibres have been studied extensively [14, 15]. However, moringa fruit pods have not been given much attention. The selection of the epoxy, as the matrix material in this research is owing to its exceptional bonding, physicochemical, thermal, mechanical, di-electrical, and aging characteristics [12, 16, 17]. Toughening epoxy material is necessary for greater impact strength, which will allow it to be used for sophisticated technical applications [18]. In research on the experimental study on mechanical properties of groundnut shell particle reinforced epoxy composite, Raju and Kamarappa [19] observed that employing groundnut shell particles as a reinforcing material in an epoxy matrix resulted in the development of a useable composite with intermediate strength. According to their investigation, the composite with 60:40 groundnut shell particles and epoxy resin percentage, and 0.5 mm particle size had the optimum tensile, bending, and impact strengths. Similar research by Oladele *et al.* [20], reported the use of particulate palm kernel shells as reinforcing elements for developing epoxy matrix composites for automobile bumper applications. Therefore, the current research was conducted to improve the characteristics of epoxy using natural reinforcements as a means of developing green and sustainable materials. Hence, wastes from the moringa oleifera fruit pod were processed as reinforcement materials in an epoxy matrix and the properties were evaluated for structural applications. Waste from moringa oleifera plant being a medicinal plant will be a human-friendly material that can be considered for biomedical application in the future. Thus, the suitability of this material for structural application will further encourage more application of MOFP.

## 2 Materials and method

### 2.1 Materials

**Figure 1:**
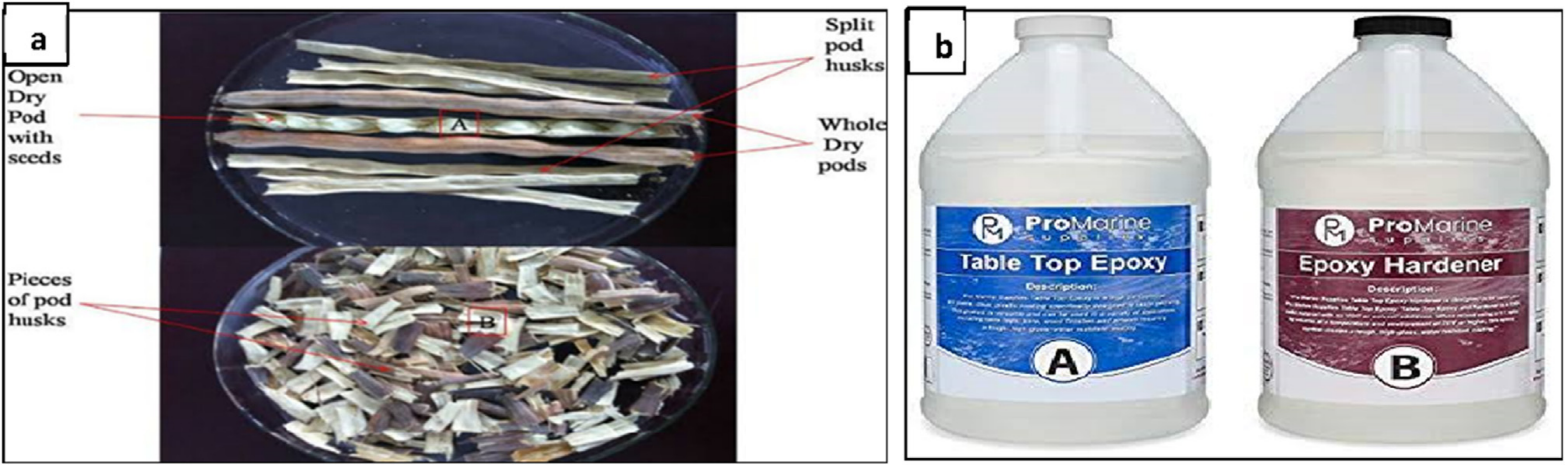
Materials (a) Sun-dried Moringa Oleifera fruit Pods (b) Epoxy resin and Hardener.

**Figure 2:**
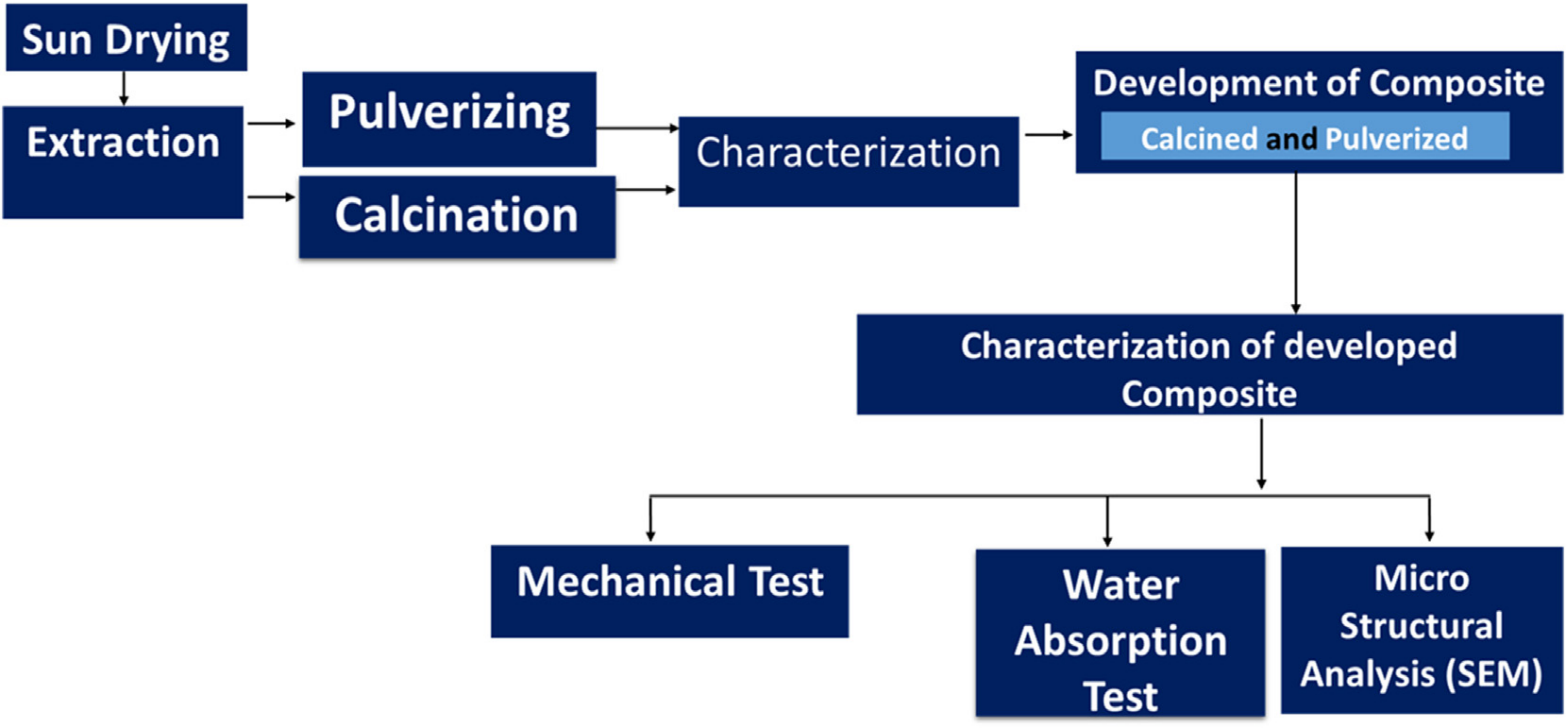
Methodology flowchart.

The Moringa oleifera fruit pods, as shown in Figure 1a, were obtained from farms in Akure, Ondo State, while the Epoxy resin and hardener, as shown in Figure 1b, were from GZ industrial supplies in Lagos, Nigeria. The flowchart for methodology is as shown in Figure 2.

### 2.2 Production of pulverized and calcined MOFP micro-particles from the moringa oleifera fruit Pod

Pulverized MOFP micro-particles were produced from the plant pod by sun-drying for 3 days after which they were pulverized via a grinding machine. Calcined MOFP was obtained by burning the pods in the furnace at 900 °C for 1 h. To obtain uniform particle sizes, the two processed particles were allowed to pass through a sieve aperture of ˂50 μm using a sieve shaker with a set of sieves.

### 2.3 Development of MOFP particulate reinforced epoxy based bio-composites

The pulverized and calcined MOFP micro-particles were integrated into the epoxy matrix in the range of 3–20 wt.% where the bio-composites were formed using the open mould technique. Respective calcined and pulverized MOFP reinforced epoxy bio-composites were formed by casting compounded materials, i.e., epoxy resins, hardener, and particulates into tensile, flexural, impact, and wear sample molds and extracted after curing. The samples after extraction from the mould were left for 30 days in the laboratory at ambient temperature for further curing. The samples were tested following appropriate ASTM standards. Table 1 illustrates the various sample formulation for the bio-composites.

**Table 1:** Formulation Ratios for developing the bio-composites.

S/N	Sample (wt.%)	Resin (g)	Hardener (g)	MOFP (g)
1	Control	100	50.0	-
2	3	97	48.5	4.5
3	6	94	47.0	9.0
4	9	91	45.5	13.5
5	12	88	44.0	18.0
6	15	85	42.5	22.5
7	18	82	41.0	27.0
8	20	80	40.0	30.0

### 2.4 Characterization and assessment of the properties of the developed bio-composites

#### 2.4.1 Flexural test

The flexural properties of the manufactured bio-composite samples were evaluated using a three-point bending experiment. The flexural test was performed in compliance with the ASTM D790-03 standard using a universal testing machine model 3369. In the machine's grasp, a sample measuring 150 × 50 × 3 mm was stretched for 65.00 mm at a 5 mm/min test speed. For each composition, three samples were examined, with the average value serving as the representative value.

#### 2.4.2 Tensile test

Tensile tests were performed on an Instron series 3369 universal testing equipment following ASTM D3039/D3039M-17 standard. The specimens utilized were dumb-bell-shaped specimens with dimensions of 90 × 10 × 3 mm. The test was performed with a 10 kg load cell and a crosshead speed of 5 mm/min. Three tests were repeatedly done for each of the produced samples to verify the accuracy and reliability of tensile test findings.

#### 2.4.3 Impact test

The notched Izod impact test was performed in accordance with ASTM D 256-10, a defined test method for testing polymer impact resistance utilizing the Izod Pendulum. The test was performed on a Hounsfield balanced impact testing equipment, serial number 3915, model number h10-3. A 64 × 11 × 3 mm impact test dimension was notched in the centre. The samples were lined down horizontally on the machine, with support lines spaced 60 mm apart. The test samples were clamped upright in a cantilever position with a V-notch at the clamp's top level. The test item was free to fall to a specified height when the machine pendulum impacted it.

#### 2.4.4 Wear test

A wear test was conducted to analyze a material's wear characteristics and establish the composite's appropriateness for a certain wear application. The samples were tested with a Taber abrasive tester model TSC-A016 following ASTM D 1044-13, a defined test procedure for transparent plastics resistance to surface abrasion. The sample's initial weight was determined before the test. After mounting each sample on the Taber abrasive tester at 150 rpm for 10 min, the weight after abrasion testing was collected. The wear samples were made with a 100 mm diameter mould with a 3 mm thickness. Eq. (1) was used to calculate the wear index of each sample:

(1) $$Wear\;index=\frac{W_{1}-W_{0}}{RPM}\;\times 1000$$

where, $W_{1}$ is the initial weight, $W_{0}$ is the final weight after surface abrasion and RPM is revolutions per minute which were 200 rpm for this test.

#### 2.4.5 Thermal test

Lee's disc apparatus was used to assess the heat conductivity of the bio-composites produced, as per ASTM E1530-19. Because the material developed was not tested at a temperature near the activation point of degradation, no temperature or thermal deterioration was found. To calculate the thermal conductivity of these samples, we used the equation in Eq. (2).

(2) $$k=\frac{m\;cp\;\left(\emptyset 1-\;\emptyset 2\right)4x}{\pi D^{2}\left(T1-T2\right)t}$$

where, k – denotes heat conductivity, m - disk mass, 7.8 × 10^−3^ kg, cp - disk specific heat capacity, 0.91 kJ/kgK (910 J/kgK), ∅ 1, ∅ 2 – starting, and final temperature of disk B, D - sample diameter, 0.04 m, x – sample thickness, 0.003 m, T1, T2 – Temperature of disk A and B in Kelvin, t = total time required to attain a constant temperature.

#### 2.4.6 Hardness test

A Shore D hardness tester was used to test the specimen in accordance with ASTM D2240-00. The samples were indented and placed on the flat surface of the tester stand. By indenting the samples in four distinct spots, four values were acquired, and the average value was used for analysis.

#### 2.4.7 Water absorption test

Water absorption test was carried out in accordance with ASTM D5229M-12, with 250 cm^3^ of water being placed in a clean plastic container. A chemical weighing balance (FA2104A model with a precision of 0.0001 g) was used to determine the dry weight of each sample, and measurements were taken every day for the next seven (7) days. Before being weighed, the samples were taken out and wiped with a neat cloth. The acquired data were used to calculate the weight gained and percent water intake using the formulas in Eqs. (3) and (4).

(3) $$W\left(g\right)=W_{t-}W_{d}$$

Eq. (3): Formula for weight gained

(4) $$\text{W ​}\left(\text{\%}\right)\text{ ​}=\frac{W_{t}-W_{0}}{W_{0}}\times 100$$

Eq. (4): Formula for percentage water absorptionWhere, W (g) is the weight gain, W (%) is percentage water absorption, $W_{d}$ and $W_{t}$ are the respective dry weight, and the weight of the sample after time t.

#### 2.4.8 SEM/EDS characterization

The EVO MA 15, Carl Zeiss SMT was used for the SEM morphological study of the particle surface, and the samples were sputtered with gold-coated sputters before the inspection to increase electrical conductivity. Furthermore, EDS investigations were carried out to establish the relative abundance of each component element. The fractured surfaces of the developed composite samples were observed through SEM that was operated at 15 kV. The samples were gold coated with a Quorum coating machine (Q150RES) to make them conductive before SEM observation.

#### 2.4.9 XRD spectrum

X-ray diffraction (XRD) patterns of the MOFP particles were obtained utilising a Bruker D2 Phaser® diffraction device with a copper K radiation source to detect the phases included in the particulate. The patterns were analyzed using the PANalytical (v3.0e) X'pert High score program and the machine was run at 30 kV and 20 mA generator settings at 25 °C.

## 3 Results and discussion

### 3.1 SEM/EDS images of the pulverized and calcined MOFP particles

**Plate 1:**
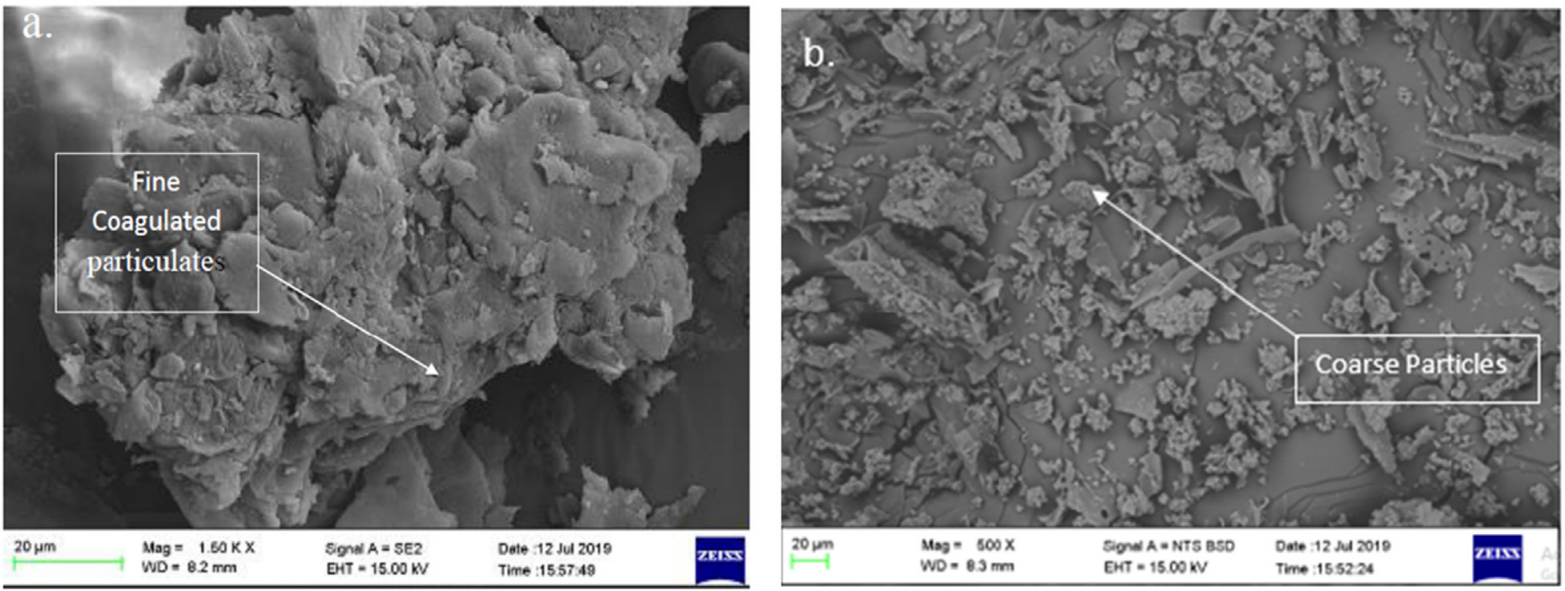
SEM image of (a) pulverized MOFP and (b) calcined MOFP.

**Plate 2:**
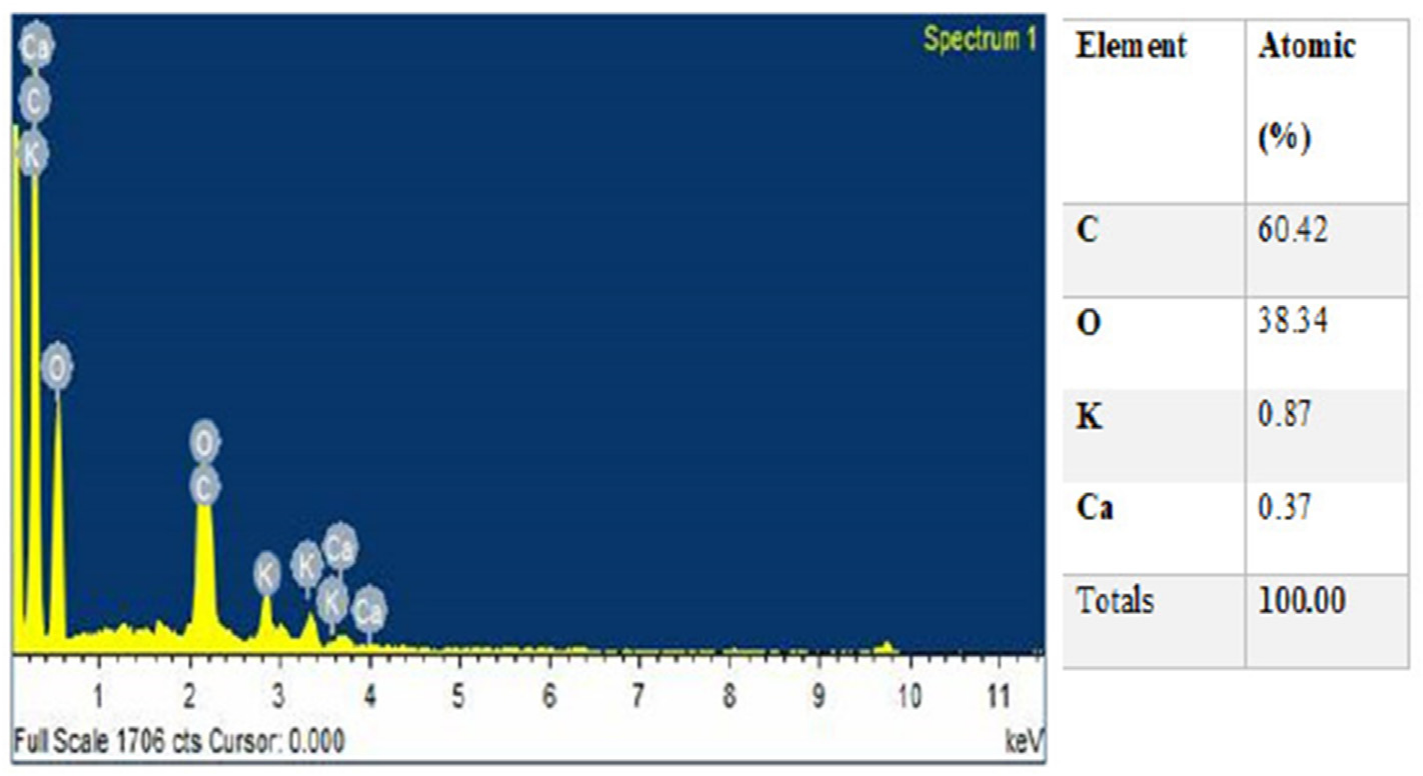
EDS spectrum of calcined MOFP with atomic % composition.

**Plate 3:**
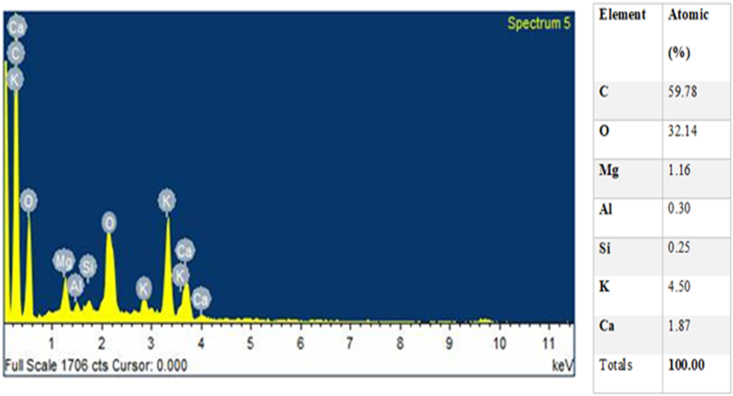
EDS spectrum of pulverized MOFP with atomic % composition.

Plates 1, 2, and 3 show the SEM/EDS images of the pulverized and calcined MOFP particles. It can be observed from Plate 2 that the calcined MOFP elemental compositions consist of Carbon (C) and Oxygen (O) in abundance (60.42 and 38.34 wt.%), respectively while Potassium (K) and Calcium (Ca) (0.87 and 0.37 wt. %, respectively) were present in a significant quantity. It can be deduced that these elements can react with one another to form ceramic oxides such as calcium oxides and potassium oxides. These oxides are strong and can enhance strength, increased wear resistance, and reduce shrinkage.

Likewise, from Plate 3, the pulverized MOFP contain Carbon (C) and Oxygen (O) in abundance (59.78 and 32.14 wt. %, respectively) and other elements like Magnesium (Mg), Aluminium (Al), Silicon (Si), Potassium (K) and Calcium (Ca) (1.16, 0.30, 0.25, 4.50 and 1.87 wt. %, respectively). Also, these elements can react with oxygen to form oxides such as Silicon Oxides, Calcium Oxide, Aluminium oxide, and Potassium Oxide and, these can enhance the mechanical, thermal, and physical properties of the reinforced materials. Generally, it was observed from Plate 3 that the pulverized MOFP consists of all the elements present in the calcined MOFP with higher atomic % composition for the major elements (Carbon (C) and Oxygen (O)) and lower atomic % composition for the minor elements (Potassium (K), and Calcium (Ca)) with additions of Magnesium (Mg), Aluminium (Al) and Silicon (Si) in variable proportions.

The presence of carbon will improve the strength, while oxygen enriches the interface strength by enhancing interface connection between macromolecular links [21]. The presence of magnesium will bring about an improvement in the flexural strength, modulus and helps in thermal stability [22]. Aluminium oxide on other hand enhances the water repellant properties [23]. Potassium may bring about an increase in strength, modulus, and fracture properties. Calcium enhances the thermal conductivity and removal of heat [24] while silicon oxide improves the abrasion resistance of the material of which they contained.

### 3.2 Flexural properties

**Figure 3:**
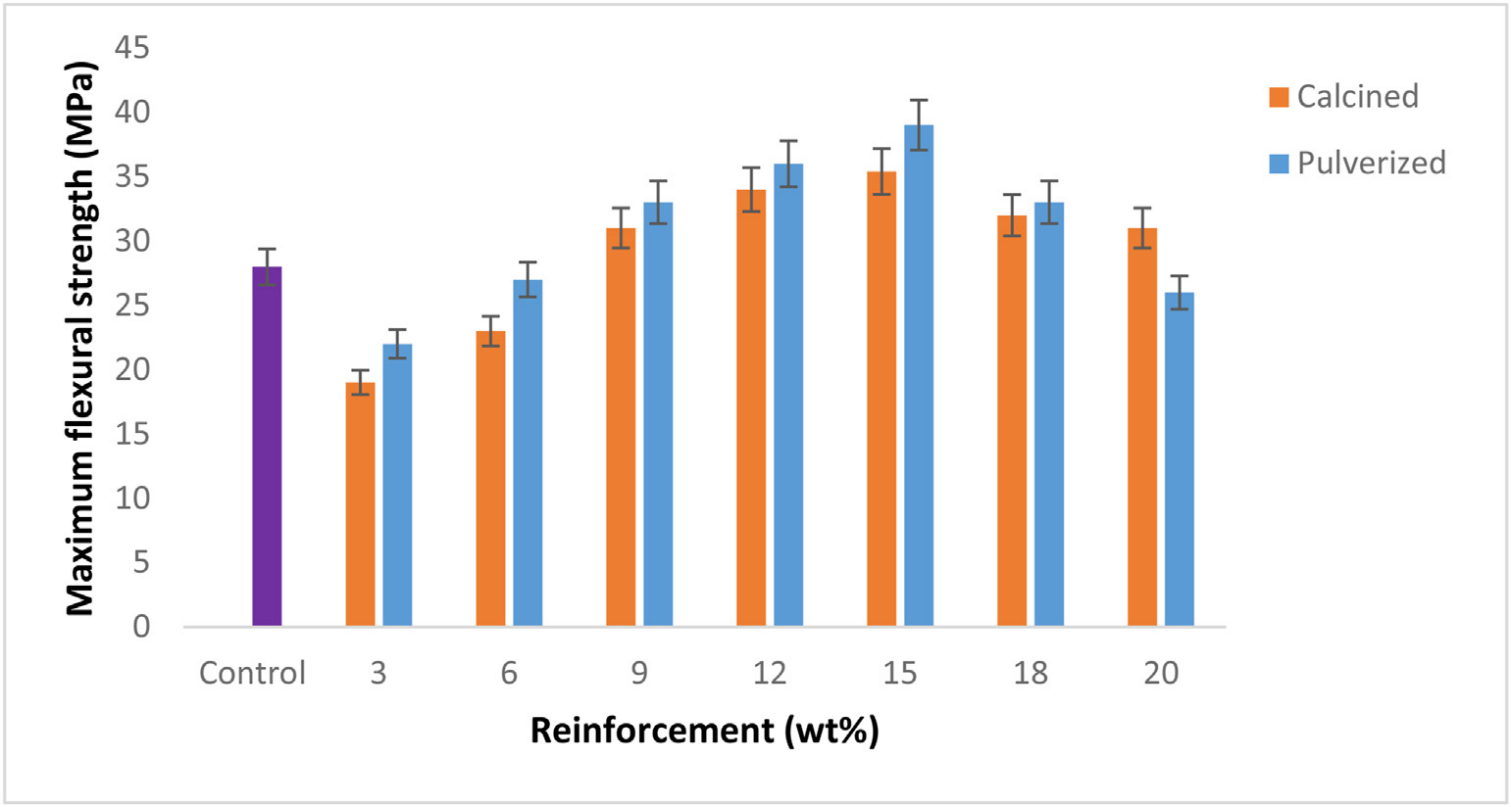
Variation of maximum flexural strength with calcined and pulverized moringa oleifera fruit Pod particles epoxy bio-composites and control.

The result of the flexural properties of calcined and pulverized moringa oleifera fruit pod particle reinforced epoxy bio-composites and the control was presented in Figure 3. The outcome shows that there was an improvement in the maximum flexural strength of the bio-composites from 9 - 15 wt. % for both calcined MOFP (CMOFP) and pulverized MOFP (PMOFP) before a decrease in the enhancement potential. The enhancement potential was noticed to be higher in PMOFP than CMOFP in the entire weight fraction used, hence, 15 wt. % pulverized MOFP reinforced epoxy bio-composite has the optimum flexural strength with a value of 39.02 MPa and 28.7 % enhancement compared to the control. This was followed by a sample from the same weight fraction for calcined MOFP having a flexural strength of 35.40 MPa which translated to about 25 % enhancement compared to the control. This could be due to the differences in surface morphologies of the MOFPs and inherent elemental compositions as revealed by the SEM/EDS in Plates 2 and 3 and excessive loading of reinforcement into the matrix which resulted into poor wetting and weak interfacial adhesion, hence, poor transfer of load from the matrix to the reinforcement. Similar to these findings were the results of Binoj, 2018 [25] where MOFP is analyzed as potential reinforcement in a polymer matrix and the optimum content was found to be 20 wt.%. and, in the work of Suherman and other researchers [26], where the flexural properties of kenaf reinforced epoxy biocomposites were studied and the biocomposite's flexural strength increased to around 130 MPa.

**Figure 4:**
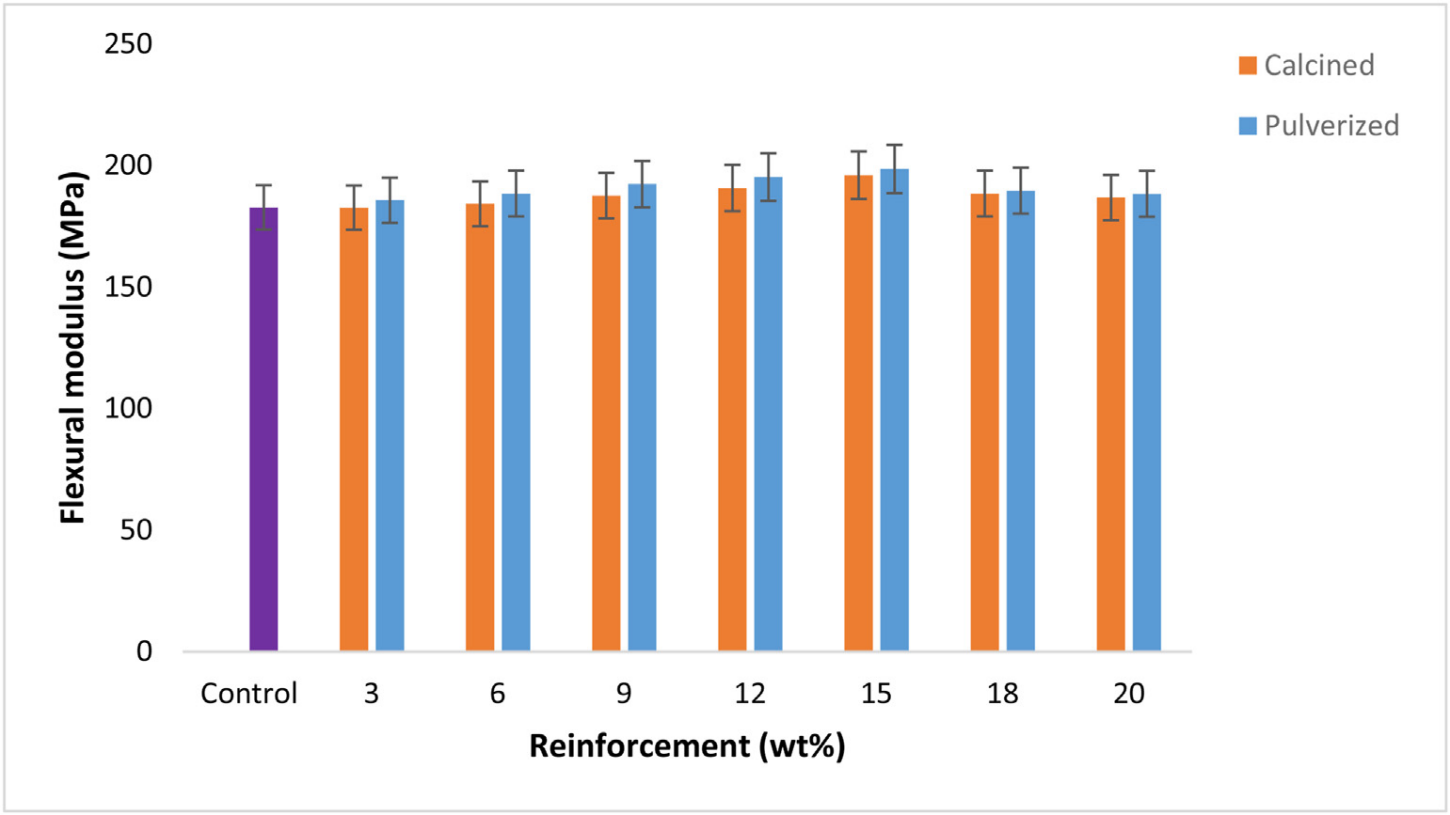
Variation of flexural modulus with calcined and pulverized MOFP reinforced epoxy bio-composites and control.

Figure 4 shows the variation of flexural modulus with calcined and pulverized MOFP reinforced epoxy bio-composites and the control. The results follow similar trends with what was obtained in Figure 3, with a slight difference in the mode of enhancement potentials from CMOFP and PMOFP. Here, the PMOFP enhancement takes effect from 6 wt. % and both MOFPs extend their potential to 20 wt. %. The enhancement potential was noticed to be higher in PMOFP than CMOFP in the entire weight fraction used, hence, 15 wt. % pulverized MOFP reinforced epoxy bio-composite has the highest flexural modulus of 198.4 MPa and 8.79 % enhancement compared to the control. This was followed by a sample from the same weight fraction for calcined MOFP having a flexural modulus of 195.4 MPa and an enhancement of about 7.2% compared to the control with a flexural modulus value of 182.6 MPa. Results of the flexural properties revealed that the flexural strengths were more improved than flexural moduli for the bio-composites.

### 3.3 Tensile properties

**Figure 5:**
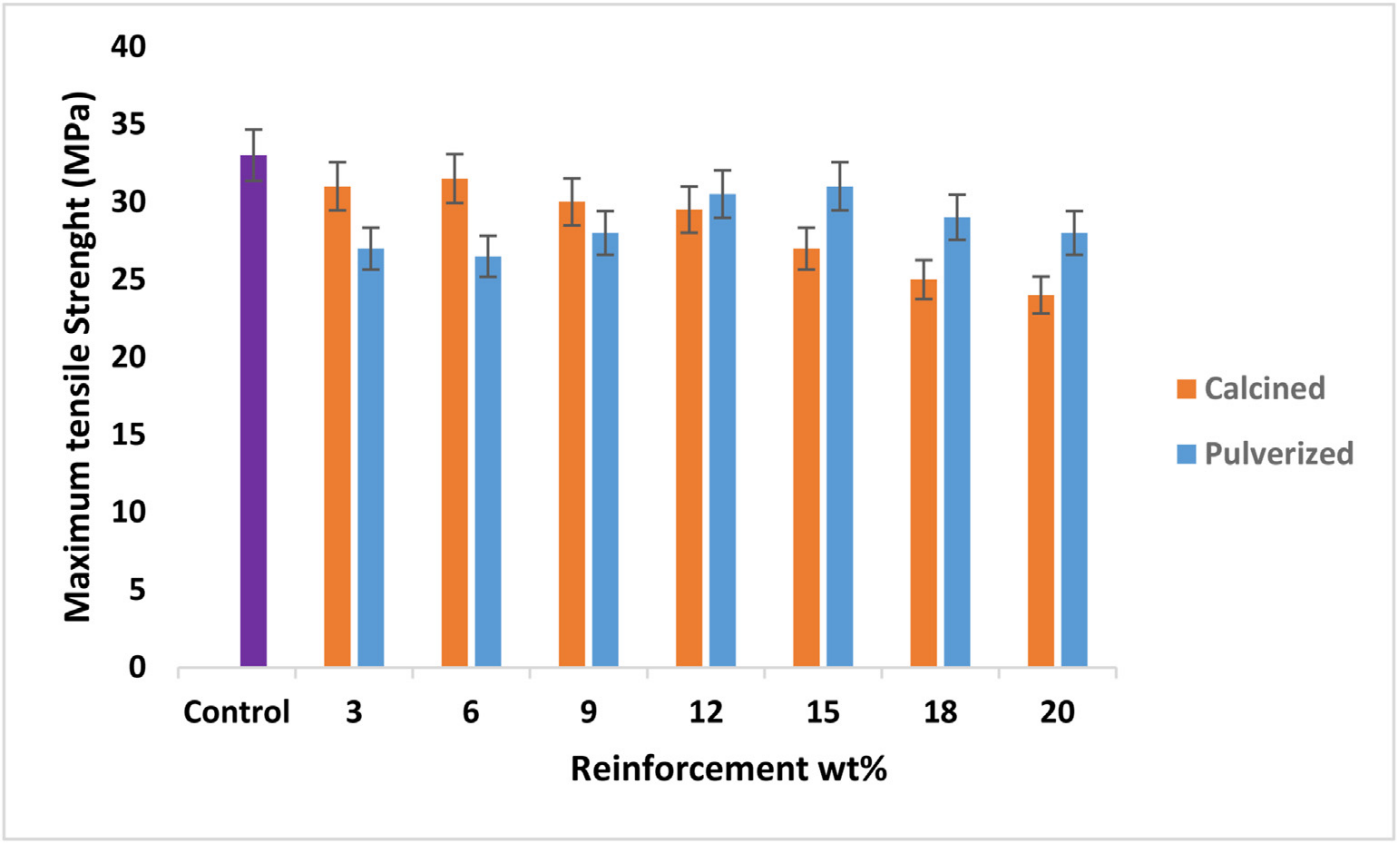
Variation of maximum tensile strength with calcined and pulverized MOFP particles reinforced epoxy bio-composites and control.

The change in maximum tensile strength between the calcined and pulverized MOFP bio-composites and the control is shown in Figure 5. In comparison to the control sample, the maximum tensile strength of the bio-composites did not improve, but rather decreased slightly. This could be attributed to a lack of appropriate tensile load transfer from the matrix to the reinforcements [27]. This indicates that the composite produced may not be suitable for tensile strength loading applications. In terms of enhancement, however, pulverized MOFP bio-composite samples outperform their calcined counterpart samples. Previous research has demonstrated that adding natural limestone fillers to a composite reduces its tensile strength when compared to an unreinforced polymer matrix because the agglomerates act as stress concentration zones in the composite [28, 29].

#### 3.3.1 Tensile modulus

**Figure 6:**
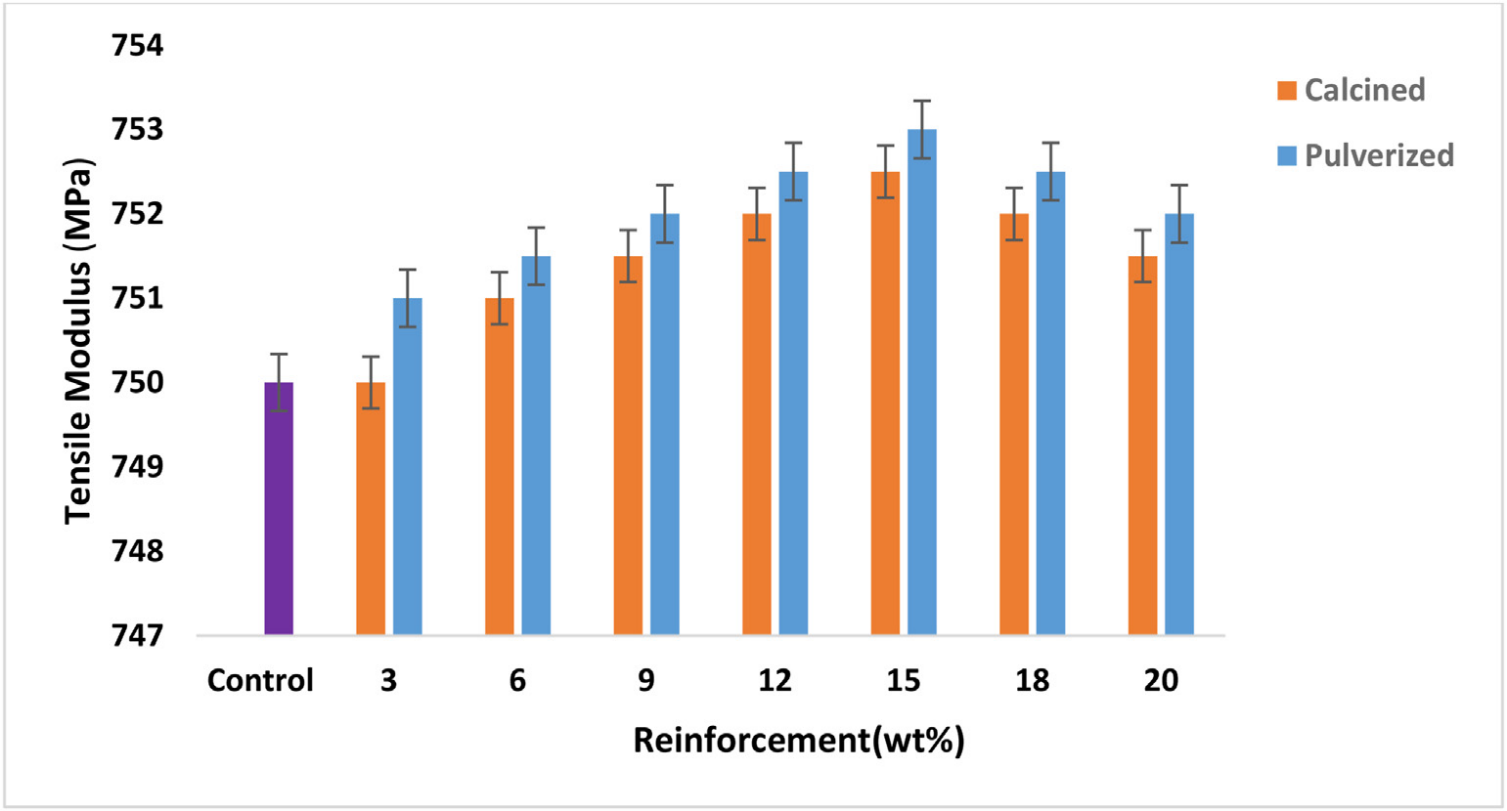
Variation of tensile modulus with calcined and pulverized MOFP particles reinforced epoxy bio-composites and control.

The variation of tensile modulus with calcined and pulverized MOFP bio-composites is shown in Figure 6. The result shows that there was an enhancement in the tensile modulus of the developed bio-composites for all the samples of calcined and pulverized MOFP except for 3 wt. % of calcined MOFP. It can also be observed that the bio-composites comprising of 15 wt. % has the optimum tensile modulus for both samples having a value of 753.28 MPa with 8.8 % enhancement and a value of 752.21 MPa with 4.8 % enhancement compared to the control for the pulverized and calcined samples, respectively. This was followed by a gradual decrease as the reinforcement content increased from 18-20 wt. %. It was further observed that the pulverized MOFP recorded a better enhancement than the calcined MOFP. The improvement observed in the tensile modulus of the bio-composite was, however, in agreement with the results of Ismail *et al.* [30], who reported that the tensile modulus of the developed composites improves with an increase in filler addition up to a certain level but decreases when the reinforcement content is at maximum. This may be due to the formation of agglomerates in the composite having higher or maximum reinforcement contents leading to the decrease observed in the tensile modulus.

### 3.4 Impact properties

**Figure 7:**
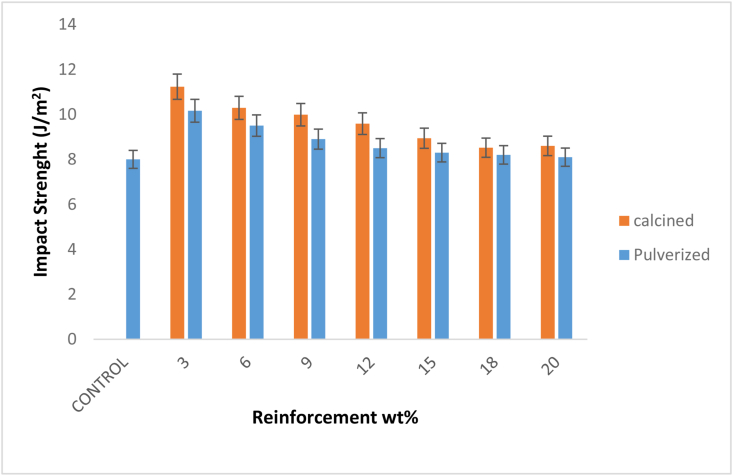
Variation of impact with calcined and pulverized MOFP particles reinforced epoxy bio-composites and control.

The change in the impact strength of the calcined and pulverized MOFP bio-composites and the control sample is shown in Figure 7. The result shows that there was a significant enhancement in the impact strength of the bio-composites for all the weight fractions for both calcined MOFP (CMOFP) and pulverized MOFP (PMOFP) when compared to the control sample. Accordingly, it was observed that the impact strength of the developed bio-composites decreases gradually as the reinforcements contents increases from 3 – 20 wt.%, and these were true for both the CMOFP and PMOFP. However, the enhancement potential was noticed to be higher in CMOFP than PMOFP in the entire weight fraction used, hence, 3 wt. % CMOFP reinforced epoxy bio-composite has the highest impact strength with a value of 11.23 J/m^2^, which is equivalent to 40.3 % enhancement compared to the control sample. This was followed by a sample from the same weight fraction for pulverized MOFP having an impact strength of 10.16 J/m^2^ that culminated to about 27 % enhancement compared to the control sample. Therefore, it was established that the addition of a particulate moringa oleifera fruit pod to the epoxy-matrix in both calcined and particulate form led to the improvement of impact strength in the bio-composites.

### 3.5 Wear properties

**Figure 8:**
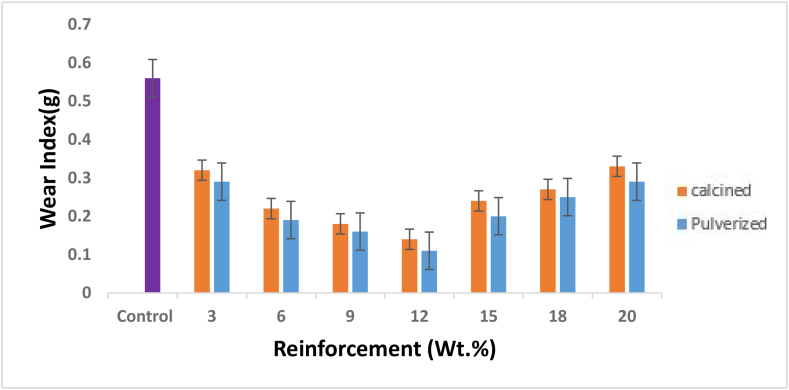
Variation of the wear rate with calcined and pulverized MOFP reinforced epoxy bio-composites and control.

Figure 8 shows that both the calcined and pulverized MOFP reinforced bio-composites had a significant improvement in their wear index. The Low friction force resulted in a lower coefficient of friction, which improved the situation. The low frictional force was attributed to the presence of natural fibre, which restricted the resin-to-abrasion-disc contact area [15, 31]. Epoxy resin wears quickly due to the exceptionally soft nature of epoxy molecules. The lower the wear index, the greater the wear resistance. Furthermore, it was also noted that for both the calcined and pulverized MOFP reinforced bio-composites, the wear index increases as the reinforcement contents increase up to 12 wt.% and then decreases for both bio-composites having reinforcement contents above 15 wt.%. Nevertheless, pulverized MOFP bio-composites show the highest improvement in wear resistance for all the weight fractions considered when compared to the calcined MOFP composites.

In general, it was observed that 12 wt.% reinforcement contents gave the optimum values for both samples i.e., pulverized MOFP yielded 80.35 % enhancement with a value of 0.11 mg while the calcined MOFP led to a 75 % enhancement with a value of 0.14 mg in wear resistance properties of the developed bio-composites.

### 3.6 Hardness properties

**Figure 9:**
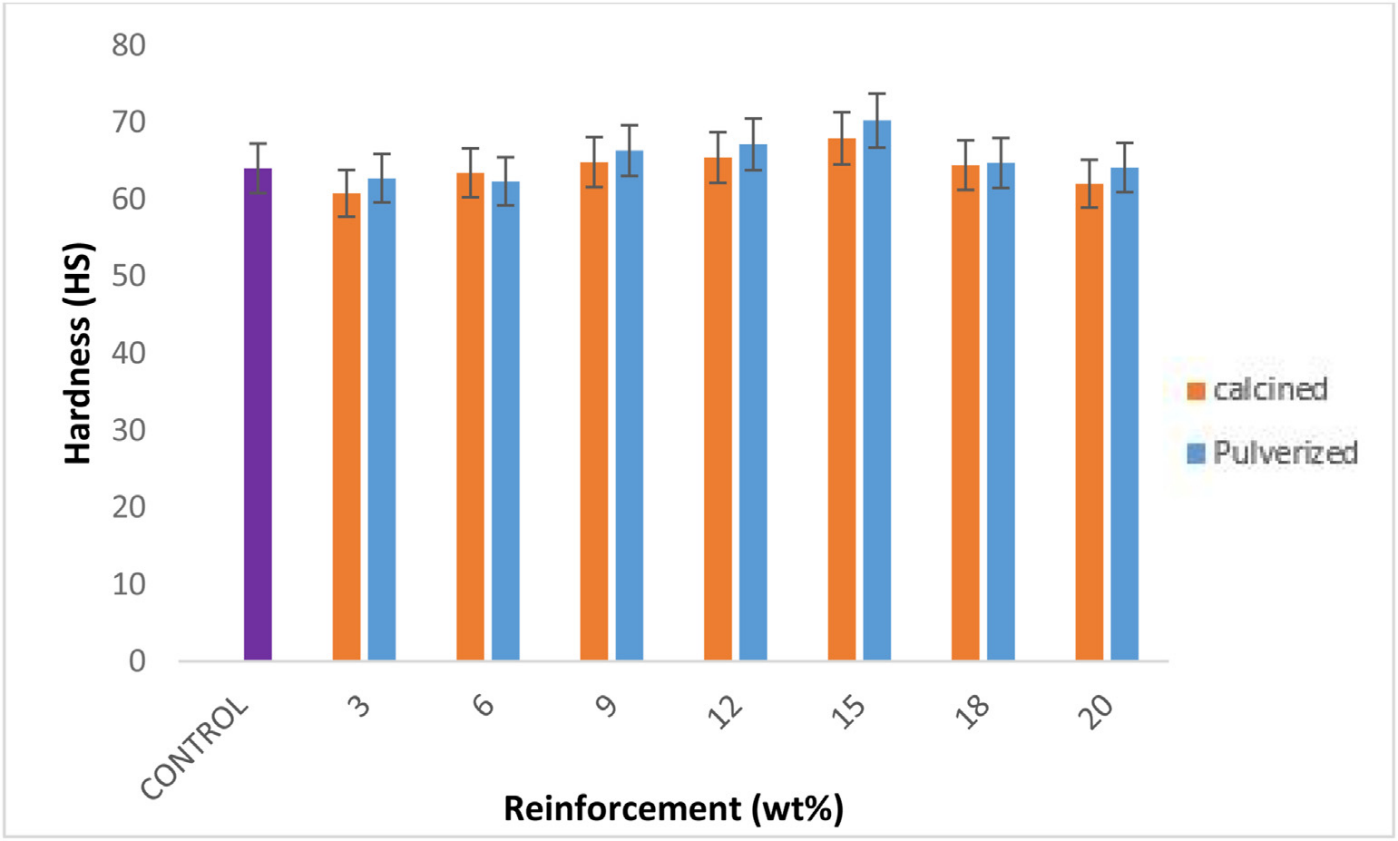
Variation of hardness with calcined and pulverized MOFP reinforced epoxy bio-composites and control.

The variation of hardness with the calcined and pulverized MOFP composites is shown in Figure 9. For the pulverized samples, an initial increase from 9-15 wt. % was followed by a gradual decrease from 18 - 20 wt. %. This trend is in agreement with Oladele *et al.* [32], in which reinforcement of polyester matrix with bagasse fiber showed an initial increase as fiber weight fraction increases followed by a decrease at higher weight fraction. Here, it was seen that the hardness was enhanced for 9, 12 and 15 wt.% pulverized MOFP composites with 15 wt. % having the highest value of 70.2 HS resulting in 67.9 % enhancement. A similar trend was observed for the calcined samples which shows an initial increase in hardness from 9 – 15 wt. % followed by a gradual constant decrease from 18 - 20 wt. %. Hence, the result showed that the hardness of the bio-composites increases with an increase in the reinforcement contents from 9 – 15 wt.% for both the calcined and pulverized MOFP reinforced bio-composites. This was in agreement with the findings of Oladele *et al.,* [32] where high density was found to improve material hardness, according to the study. When both calcined and pulverized MOFP reinforced bio-composites were compared based on their hardness properties, the pulverized samples had better characteristics.

### 3.7 Thermal properties

**Figure 10:**
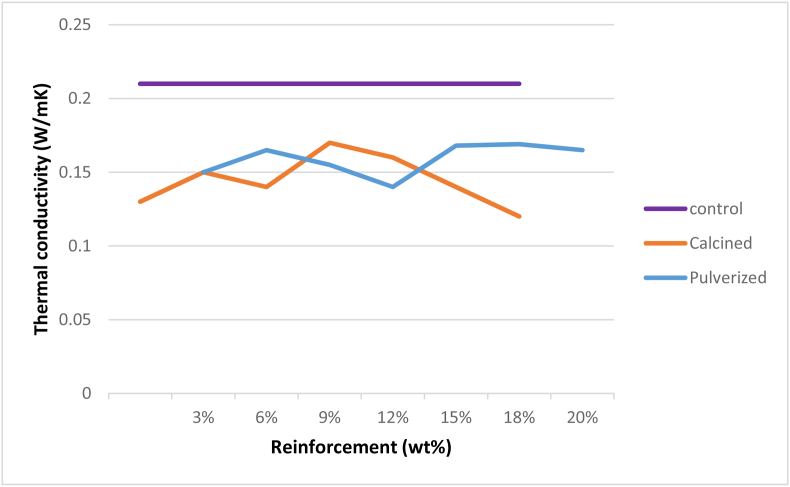
Variation of thermal conductivity with calcined and pulverized moringa oleifera fruit pod reinforced epoxy bio-composites and control.

Figure 10 shows the variation of thermal conductivity of the developed bio-composites with increasing calcined and pulverized MOFP reinforcement contents. All the developed bio-composites showed a lower thermal conductivity than the control sample. The result shows that there was a notable decrease in the thermal conductivity of the bio-composites for all the weight proportions for both calcined MOFP (CMOFP) and pulverized MOFP (PMOFP), this reduction in the thermal conductivity of the developed bio-composites could be due to the introduction of the MOFP particles which has altered the property of the thermally stable epoxy resin. However, when both the developed bio-composites were compared, the enhancement potential was noticed to be higher in CMOFP than PMOFP in the entire weight fraction considered, this could be due to the variation in the elemental constituents of the calcined and pulverized samples as revealed by the EDS results in Plates 2 and 3 respectively, hence, 12 wt.% calcined MOFP reinforced epoxy bio-composite gave the optimum thermal conductivity with a value of 0.17 W/mK compared to its counterpart reinforced with pulverized MOFP with a value of 0.14 W/mK. This implies that the addition of the reinforcement enhanced the thermal property by reducing its conductivity because thermal conductivity is a measure of how well a material can resists temperature fluctuations. This was in agreement with the findings of Nayak, *et al.* [33], that the thermal conductivity of the fabricated bio-composites decreases with the increase of filler addition thereby improving its thermal insulation capability.

### 3.8 Water absorption properties

**Figure 11:**
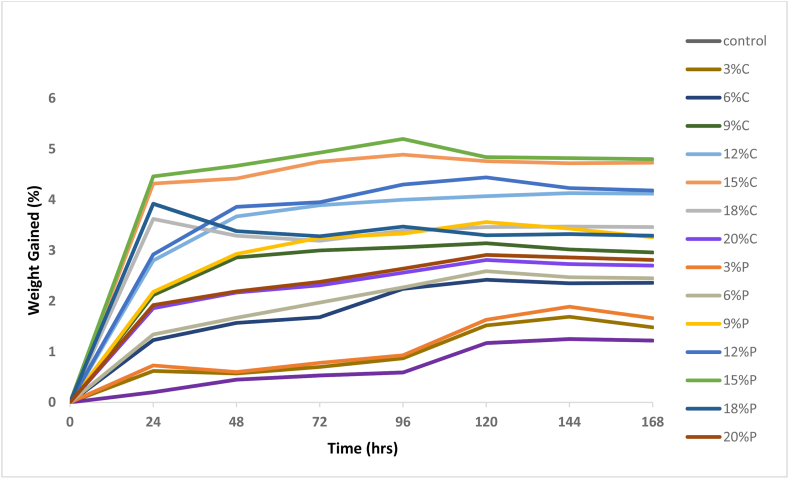
Variation of percentage weight gain per 24 h for control, calcined and pulverized MOFP reinforced epoxy bio-composites.

It was observed from Figure 11 that the calcined MOFP reinforced epoxy bio-composites has better water absorption property than the pulverized bio-composites. This may be due to the carbonization process carried out on the calcined bio-composites which reduces the hydrophilic property of the bio-composites. From the graph, all the bio-composites absorb water very rapidly and linearly at the initial stage after which the saturation level was attained at 144 h without any further increase in water absorption.

According to Fick's law, which measures the amount of material that will flow across a unit area in a unit time interval, water diffusion in polymer occurred in this manner. Because water is a transmission channel for oxygen and ions, understanding how water diffuses in polymers is crucial [34, 35, 36]. Fick's law, which describes the diffusion path and relaxation conditions, is the first step in understanding water diffusion. Due to the intricacy of polymer structure and compositions, as well as the difficulties of interpreting molecular-level data for such a small sample, this is complex.

The second assumption of ideal Fick's diffusion is that polymer change due to water diffusion is limited, which in most cases is unacceptably low. Frisch asserts that three forms of water diffusion are conceivable depending on the specific rate of polymer relaxation compared to water diffusion [37]. In the results shown in Figure 9, these three kinds of water diffusion were identified.

The rate of diffusion from 0 to 24 h was congruent with Case I (n = 0.5), in which polymer relaxation occurs far quicker than water diffusion and is followed by an immediate system response, culminating in Fick's behaviour. The system's rapid reactivity necessitates a high degree of flexibility in the polymer chains, i.e., a rubbery state of the polymer; in this case, the diffusion coefficient regulates diffusion [35].

Case II (n = 1) and Super Case II (Irregular diffusion, n > 1) both agreed on the diffusion rate from 28 to 120 h. A rate of diffusion that is much quicker than that of initially relaxing defines the ultimate extent of water diffusion, which marks the boundary between the (stressed equilibrium) expanding gel and the glassy core of polymers. Polymers begin to swell at this moment. Then, a rate of diffusion equal to that of relaxation (Irregular diffusion, n > 1), where the evolution of Super Case II converting from Case II makes it rather suspect in some circumstances [35, 36, 37, 38]. The final phase of these studies, which lasted from 144 to 168 h, showed that water diffusion or absorption became steady or stable, which is known as the saturation point.

For the calcined and pulverized bio-composites, the water absorption tends to increase with an increase in the moringa oleifera fruit pod content except for 18–20 wt.% which has water uptake of 3.46 and 3.29 for 18 wt.% of calcined and pulverized samples respectively and water uptake of 2.7 and 2.81 for 20 wt.% of calcined and pulverized samples respectively.

The total moisture uptake of the control sample after 168 h was 1.25%. This implies that the moringa oleifera fruit pod has an affinity for water and therefore, increased the water absorption more than that of control. This is due to the hydrophilic nature of these reinforcements which culminated in the high-water uptake observed in the developed epoxy-based bio-composite.

### 3.9 Scanning electron microscopy

**Plate 4:**
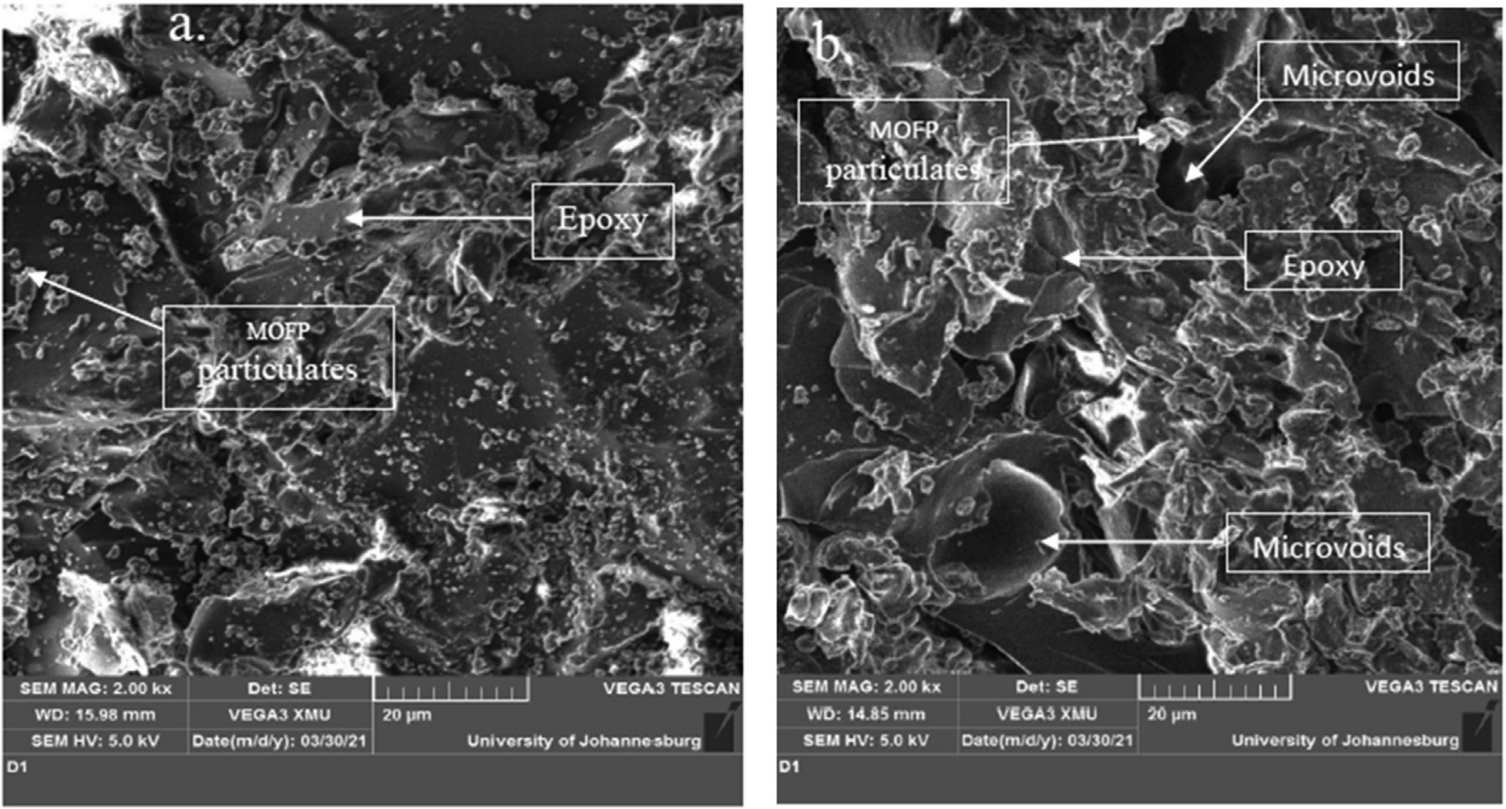
SEM Images of 3 wt.% (a) Calcined MOFP, (b) Pulverized MOFP Reinforced Epoxy Composite.

**Plate 5:**
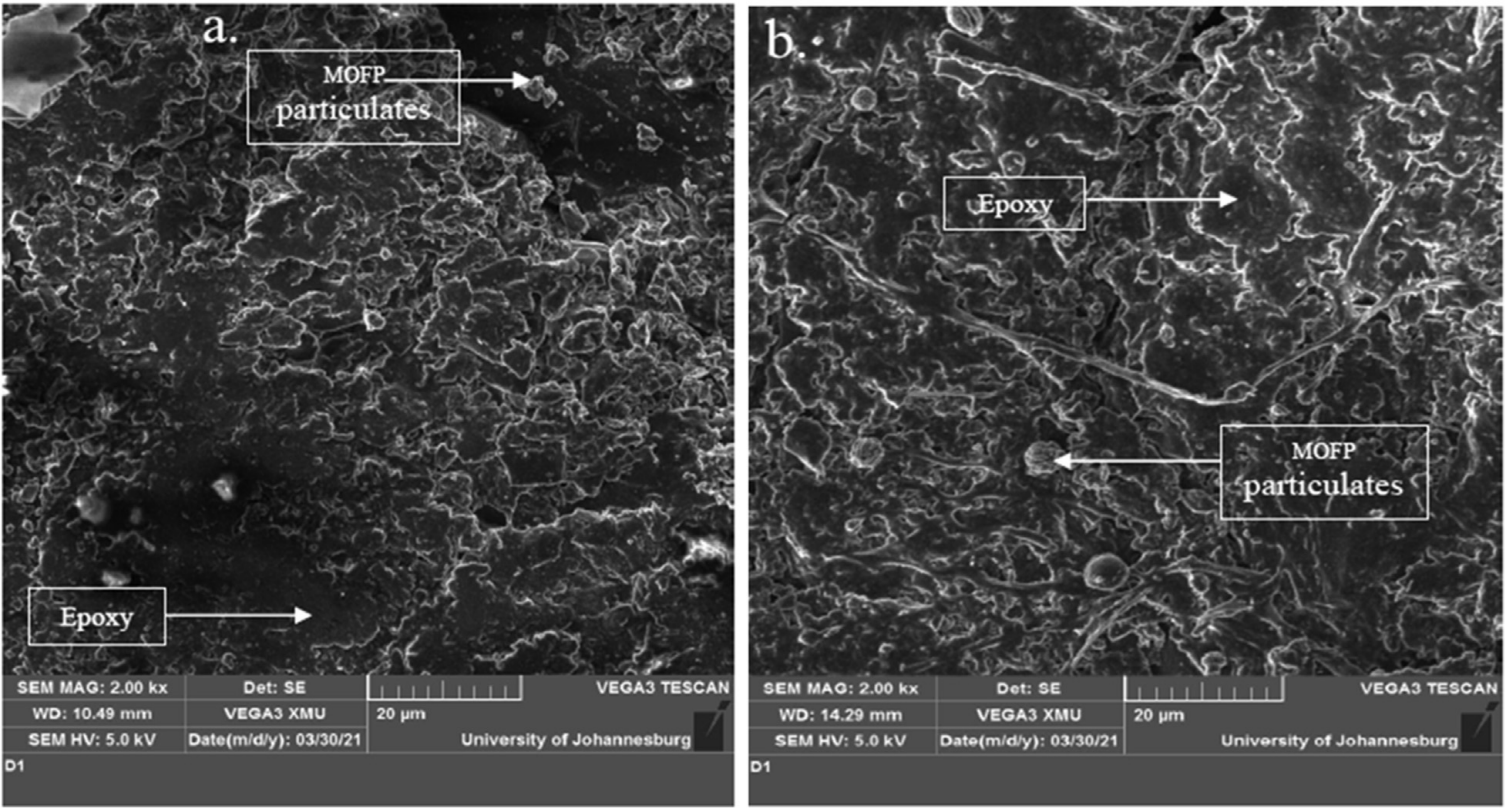
SEM Images of 9 wt. % (a) Calcined MOFP, (b) Pulverized MOFP Reinforced Epoxy Composite.

**Plate 6:**
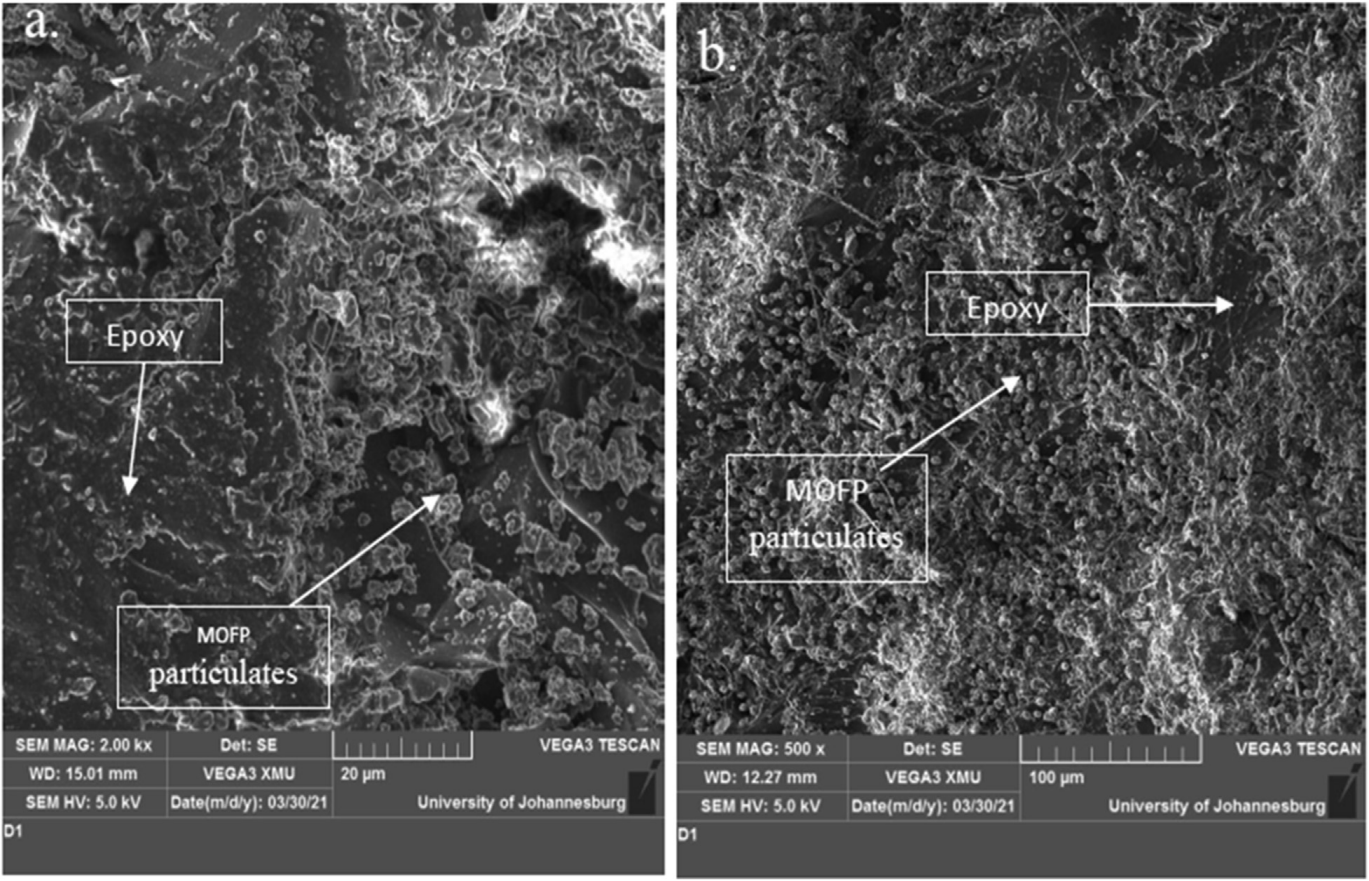
SEM Image of 15 wt. % (a) Pulverized MOFP, (b) Calcined MOFP Reinforced Epoxy Composite.

Plates 4, 5 and 6 showed the SEM images of the fractured surfaces of 3, 9, and 15 wt.% pulverized and calcined MOFP composites, respectively with certain features being identified in the images. The micrographs showed a good interface and a uniform distribution of the reinforcements in the matrix. Plate 4(a) shows the presence of microvoids which tends to reduce the strength of the composite and may be responsible for the decrease in the flexural properties observed at 3 wt.% while Plate 4(b) revealed quite uniform distribution of the reinforcements in the matrix.

Plates 5(a) and (b) also showed comparable characteristics to Plate 4, with a more noticeable distribution of reinforcements in the epoxy matrix and no microvoids. As a result, its performance was intermediate and satisfactory when compared to those in Plate 4.

Plates 6(a) and (b) also showed the dispersion of pulverized and calcined moringa particles. The reinforcements in Plate 6(a) were well disseminated in the epoxy matrix, with no evidence of microvoids. Plate 6(b) showed that the reinforcements in the matrix were dispersed rather evenly. As a result, the sample emerged as the overall best sample in most of the parameters evaluated, displaying strong interface bonding and rather uniform distribution of the reinforcements in the matrix.

### 3.10 XRD analysis of calcined and pulverized MOFP particulate reinforced epoxy biocomposites

**Plate 7:**
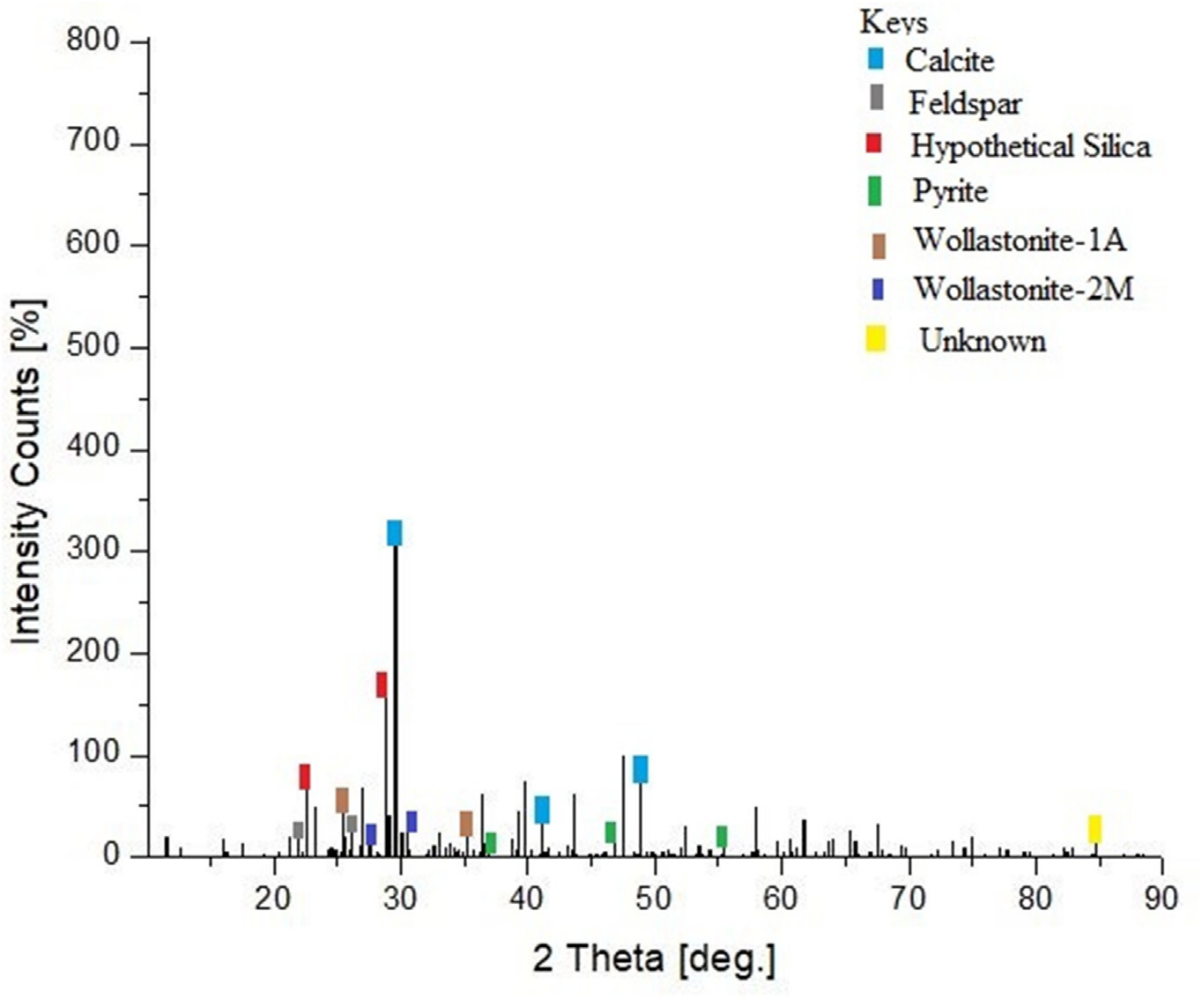
XRD Pattern of the calcined MOFP particles-epoxy based biocomposites.

**Plate 8:**
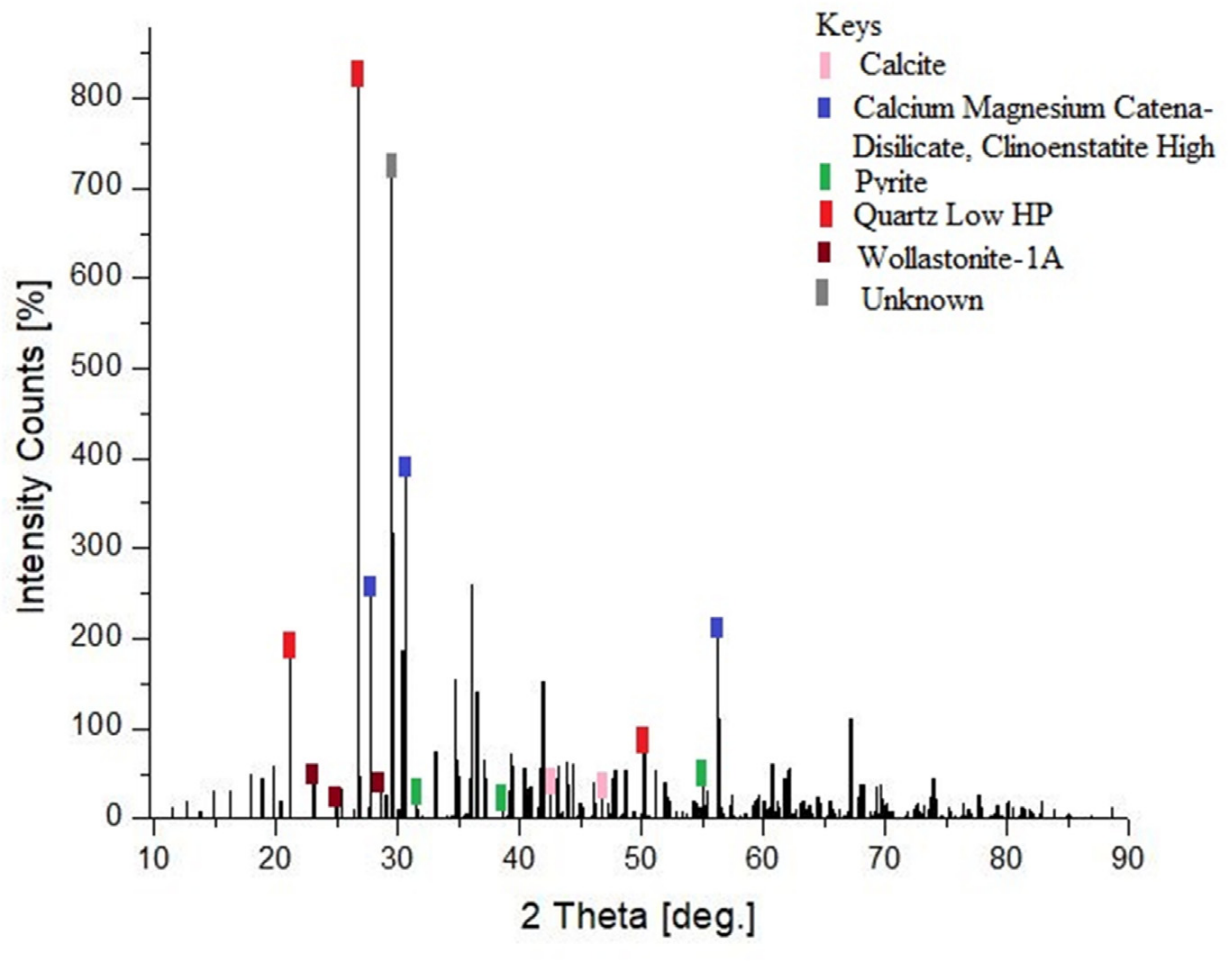
XRD Pattern of the pulverized MOFP particles-epoxy based biocomposites.

The x-ray diffraction peaks acquired for the calcined MOFP particles-epoxy-based bio-composite were as shown in Plate 7. The diffraction patterns indicated a crystalline phase revealing the main constituent of the calcined MOFP particles-epoxy based bio-composite to be calcium carbonate phase in the form of calcite (CaCO_3_). The main XRD intensity peak noticed for the calcined MOFP particles-epoxy-based bio-composite at 2θ angle is 21.81°. Similar results were also reported in the literature [39, 40]. The XRD patterns of the pulverized MOFP particles-epoxy-based bio-composite were as shown in Plate 8. The result showed that the majority of the patterns validate the existence of SiO_3_ in the form of Quartz Low HP. The main XRD intensity peak discovered for the pulverized MOFP particles-epoxy-based bio-composite at a 2θ angle is 19.55°. These results agreed with the analysis of the SEM/EDS of the MOFP particles in Plates 1, 2, and 3. The elemental compositions of the respective particulates of MOFP were responsible for the compounds formed as identified by XRD analysis of the developed MOFP particles-epoxy-based biocomposites.

## 4 Conclusions

The need for more environmentally acceptable green and sustainable materials has contributed to the selection of particulate moringa oleifera waste pods for the development of epoxy-based biocomposites that can be suitably used for structural applications. Moringa plant has found relevant applications as a medicinal plant, hence, the need to find more applications for its by-product that is currently regarded as waste. From the research carried out and the analysis of various results, it was discovered that; MOFP particles contain Carbon (C) Oxygen (O) Calcium (Ca), and Potassium (K) in both pulverized and calcined form while the pulverized particles were observed to possess some additional elements like Aluminum (Al), Magnesium (Mg) and Silicon (Si). The mechanical and thermal properties of the developed bio-composites were particularly improved with pulverized MOFP particulates addition which may be due to the presence of more elements in the pulverized particles and, in higher proportions compared to the calcined particles. It gave enhancement in flexural strength (28.7%), flexural modulus (25%), tensile modulus (8.8%), and hardness (67.9%). The optimum weight fraction for the utmost improved properties of the developed composites from pulverized MOFP microparticles was from 15 wt%. However, there was no improvement in the tensile strength of the composite samples for both reinforcements which showed that the resulting composites may not be suitable in areas of application where tensile strength is a major requirement. The benefits of using this plant-based mate could also be derived from its medicinal applications since the pulverized fruit pods contain more elemental compositions compared to the calcined fruit pods.

## Declarations

### Author contribution statement

Isiaka Oluwole Oladele: Conceived and designed the experiments; Analyzed and interpreted the data; Wrote the paper.

Gabriel Seun Ogunwande: Performed the experiments; Analyzed and interpreted the data.

Anuoluwapo Samuel Taiwo: Analyzed and interpreted the data; Wrote the paper.

Senzeni Sipho Lephuthing: Contributed reagents, materials, analysis tools or data.

### Funding statement

This work was supported by African Materials Science and Engineering Network (A Carnegie-IAS RISE network) and the DST-NRF Centre of Excellence in Strong Materials (ARPDF 1803).

### Data availability statement

Data included in article/supplementary material/referenced in article.

### Declaration of interests statement

The authors declare no conflict of interest.

### Additional information

No additional information is available for this paper.

## References

[1] Amine L.S. (2003). An integrated micro-and macro-level discussion of global green issues: ‘it isn’t easy being green. J. Int. Manag..

[2] Oladele I.O., Omokafe M.S., Olusegun J.S. (2016). Influence of chemical treatment on the constituents and tensile properties of selected agro-fibres. West Indian J. Eng..

[3] Baillie C. (2003). Editorial eco-composites. Compos. Sci. Technol..

[4] Oladele I.O., Aliu S.O., Taiwo A.S., Agbeboh N.I. (2022). Comparative investigation of the influence of stone-dust particles and bagasse fiber on the mechanical and physical properties of reinforced recycled high-density polyethylene bio-composites. Compos., Adv. Mater..

[5] Bismarck A., Baltazar-Y-Jimenez A., Sarikakis K. (2006). Green composites as panacea? Socio-economic aspects of green materials. Environ. Dev. Sustain..

[6] Oladele I.O., Omotosho T.F., Ogunwande G.S., Owa A.F. (2021). A Review on the philosophies for the advancement of polymer-based composites: past, present and future perspective. Appl. Sci. Eng. Progress.

[7] Barari B., Omrani E., Dorri Moghadam A., Menezes P.L., Pillai K.M., Rohatgi P.K. (2016). Mechanical, physical and tribological characterization of nano-cellulose fibers reinforced bio-epoxy composites: an attempt to fabricate and scale the ‘Green’ composite. Carbohydr. Polym..

[8] Shuhua W., Qiaoli X., Fen L., Jinming D., Husheng J. (2013). Preparation and properties of cellulose-based carbon microsphere/poly (lactic acid) composites. J. Compos, Mater..

[9] Milanese A.C., Cioffi M.O.H., Voorwald H.J.C. (2011). Mechanical behavior of natural fiber composites. Procedia Eng..

[10] Oladele I.O., Makinde-Isola B.A., Adediran A.A., Ayanleye O.T., Taiwo S.A. (2021). Influence of structural physiognomies of pawpaw fiber–glass fiber hybrid–based green composites on mechanical properties and biodegradation potential of epoxy composites. J. Reinforc. Plast. Compos..

[11] Agbabiaka O.G., Oladele I.O., Olorunleye P.T. (2014). Investigating the influence of alkalization on the mechanical and water absorption properties of coconut and sponge fibers reinforced polypropylene composites. Leonardo Electron. J. Pract. Technol..

[12] Abdul D.A.S. (2007).

[13] Ahmad F., Choi H.S., Park M.K. (2015). A review: natural fiber composites selection in view of mechanical, light weight, and economic properties. Macromol. Mater. Eng..

[14] Shalwan A., Yousif B. (2012). In State of Art: mechanical and tribological behaviour of polymeric composites based on natural fibres. Mater. Des..

[15] Shalwan A., Yousif B. (2014). Influence of date palm fibre and graphite filler on mechanical and wear characteristics of epoxy composites. Mater. Des..

[16] Islam T., Nigar F., Saha S., Tapash A., Sharmin N., Dey K., Mustafa A. (2011). Fabrication and mechanical characterization of jute fabrics: reinforced polyvinyl chloride/polypropylene hybrid composites. Appl. Sci. Eng. Progress.

[17] Rozman H., Musa L., Abubakar A. (2005). The mechanical and dimensional properties of rice husk-unsaturated polyester composites. Polym. Plast. Technol. Eng..

[18] Kumar M., Gowkanapalli R.R., Rao H., Reddy K., Reddy B. (2012). Assessment of glass/drumstick fruit fiber (moringa oleifera) reinforced epoxy hybrid composites. Appl. Sci. Eng. Progress.

[19] Raju G.U., Kumarappa S. (2011). Experimental study on mechanical properties of groundnut shell particle reinforced epoxy composite. J. Reinforc. Plast. Compos..

[20] Oladele I.O., Taiwo A.S., Daramola O.O., Akinwekomi A.D., Agbabiaka O.G. (2019).

[21] Zhao Z., Mu X., Wu J., Qi H.J., Fang D. (2016). Effects of oxygen on interfacial strength of incremental forming of materials by photopolymerization. Extreme Mech. Lett..

[22] Kiaei M., Moghdam Y., Kord B., Samariha A. (2017). The effect of Nano-MgO on the mechanical and flammability properties of hybrid nano composites from wood flour-polyethylene. Maderas Cienc. Tecnol..

[23] El-Meligy M.G., Nagieb Z.A., Isis K.B. (2012). Effect of variation Aluminum oxide concentration on the modified novolac stalk composite. ISRN Chem. Eng..

[24] Rothon R., Rothon R. (2017). Particulate Fillers in Thermoset Plastics BT - Fillers for Polymer Applications.

[25] Binoj J.S. (2018). Characterization and optimization of mechanical properties of sustainable moringa oleifera fruit husk fiber for polymer composite applications. SAE Techn. Paper.

[26] Suherman H.E., Azwar, Duskiardi Yovial, Septe E. (2019). Properties of KenafFibers/epoxy biocomposites: flexural strength and impact strength. Mater. Sci. Eng., A.

[27] Kolawole S., Omoniyi A. (2019). Preparation and characterization of epoxy filled snail shell thermoset composite. Direct Res. J. Chem. Mater. Sci..

[28] Boronat T., Fombuena V., Garcia-Sanoguera D., Sanchez-Nacher L., Balart R. (2015). Development of a biocomposite based on green polyethylene biopolymer and eggshell. Mater. Des..

[29] Owuamanam I.S. (2019).

[30] Ismail N.I., Ishak Z.A.M. (2018). Effect of fiber loading on mechanical and water absorption capacity of Polylactic acid/Polyhydroxybutyrate-co-hydroxyhexanoate/Kenaf composite. IOP Conf. Ser. Mater. Sci. Eng..

[31] Ramesh M., Atreya T.S.A., Aswin U.S., Eashwar H., Deepa C. (2014). Processing and mechanical property evaluation of banana fiber reinforced polymer composites. Procedia Eng..

[32] Oladele I.O. (2014). Effect of bagasse fibre reinforcement on the mechanical properties of polyester biocomposites. J. Assoc. Prof. Eng. Trinidad Tobago.

[33] Nayak R., Tarkes D.P., Satapathy A. (2010). A computational and experimental investigation on thermal conductivity of particle reinforced epoxy composites. Comput. Mater. Sci..

[34] Hickner M. (2012). Water-mediated transport in ion-containing polymers. J. Polym. Sci. B Polym. Phys..

[35] Yang C., Xing X., Li Z., Zhang S. (2020). A comprehensive review on water diffusion in polymers focusing on the polymer–metal interface combination. Polymers.

[36] Pathania A., Arya R., Ahuja S. (2017). Crosslinked polymeric coatings: preparation, characterization, and diffusion study. Prog. Org. Coating.

[37] Crank J. (1967).

[38] Marais S., Hirata Y., Cabot C., Morin-Grognet S., Garda M.-R., Atmani H., Poncin-Epaillard F. (2016). Effect of a low-pressure plasma treatment on water vapor diffusivity and permeability of poly(ethylene-co-vinyl alcohol) and polyethylene films. Surf. Coating. Technol..

[39] Nayak S., Kumar Khuntia S. (2019). Development and study of properties of Moringa oleifera fruit fibers/polyethylene terephthalate composites for packaging applications. Compos. Commun..

[40] Rahman M., Netravali A., Tiimob B., Rangari V. (2014). Bioderived ‘green’ composite from soy protein and eggshell nanopowder. ACS Sustain. Chem. Eng..

